# A Review of Commercially Available Point-of-Care Devices to Concentrate Platelet-Rich Plasma

**DOI:** 10.7759/cureus.28498

**Published:** 2022-08-28

**Authors:** Toufic R Jildeh, Charles A Su, Matthew L Vopat, Justin R Brown, Johnny Huard

**Affiliations:** 1 Orthopedic Surgery, Michigan State University, East Lansing, USA; 2 Orthopedic Surgery, The Steadman Clinic, Vail, USA; 3 Biological Sciences, The Steadman Clinic, Vail, USA

**Keywords:** point-of-care biologics, point-of-care, orthobiologics, biologics, platelet rich plasma

## Abstract

Platelet-rich plasma (PRP) is a promising therapy treatment option for multiple orthopedic conditions, which has demonstrated expanding clinical use. With increased clinical use of PRP, there has been a greater demand for point-of-care (POC) biologic devices. For this review, publicly available information provided by the device corporations, PubMed, Medline, and Embase databases were searched for studies related to POC device function. A scoping review study design was selected to explore the breadth of knowledge in the literature regarding PRP POC devices. ProofPoint Biologics demonstrated the highest laboratory platelet increase (5.2 ± 0.28-fold) and the longest processing time (49 ± 1.4 minutes). Celling demonstrated the lowest laboratory platelet increase (2.7 ± 0.8-fold), while AcCELLerated had the fastest processing time (18 ± 1.4 minutes for PurePRP® AB60 Pure (Pure Accelerated Biologics, Tequesta, FL) and 13.5 ± 2.1 minutes for AbsolutePRP® (Emcyte Corporation, Fort Myers, FL)). Celling had the lowest cost out of the various biologic devices. There is significant variability in the technical features, cost, processing time, and centrifugation parameters of the different commercially available point-of-care devices.

## Introduction and background

Platelet-rich plasma (PRP) is an autologous derivative of whole blood and is perhaps the most widely used in biologic treatment modality over the last two decades [1-4]. PRP use was first described in oral maxillofacial and cardiac surgery in the 1980s, and its use in orthopedic surgery has increased exponentially [1]. PRP has been used to treat numerous musculoskeletal pathologies, from joint arthridities to tendon/ligament pathologies. The therapeutic effect of PRP is initiated by platelet activation, whereby they release various cytokines and growth factors that have positive effects on cell proliferation, angiogenesis, cell chemotaxis, and matrix synthesis [5-11]. The numerous growth factors that are released include transforming growth factor-beta (TGF-β), fibroblast growth factor (FGF), platelet-derived growth factor (PDGF), vascular endothelial growth factor (VEGF), insulin-like growth factor (IGF-1), epithelial growth factor, and connective tissue growth factor [10,12]. The goal of PRP treatment is to provide damaged tissue with higher concentrations of these cytokines and growth factors to decrease inflammation and promote physiologic healing.

PRP is generated by centrifugation, and while all platelet concentrations above physiologic levels are considered PRP, commercial PRP kits can produce concentrations that range significantly from 0.52× to 9× baseline levels depending on the product used [1]. While the ideal concentration of platelets to optimally treat various diagnoses remains to be fully defined, current evidence suggests concentrations of 2.5× to 6× baseline may be optimal, with concentrations greater than 10× showing potentially slower healing [1,6]. Formulations of PRP may be further classified as leukocyte-rich PRP, defined as a leukocyte concentration above baseline, and leukocyte-poor PRP, defined as a leukocyte concentration below baseline [8].

With increased clinical use, there has been an increased demand for point-of-care (POC) biologic devices for PRP cultivation. There is significant variability in POC devices in the market regarding platelet concentrating ability, processing time, and price. The purpose of this article is to provide our experience with POC PRP devices and provide a comprehensive overview of the concentration ability, processing time, and price of several commercially available POC devices.

## Review

ProofPoint Biologics manual method

The ProofPoint Biologics manual method is a completely non-automated transfer of blood products using an open pipet system [3]. Compared to the other systems described in this review, it is quite time-sensitive for the technician due to this system being completely manual. For each system, the final concentration was blinded from the single technician used for the duration of the study. After the patient’s venipuncture of 60 mL, 30 mL syringes are taken under a biosafety hood, and 1.0 mL of the whole blood product is added to a 2.0 mL microcentrifuge tube to perform a hematology analysis with the Cell-Dyn 3700 (Abbott Laboratories (Diagnostics Division), Abbott Park, IL) [9]. A complete blood count is quantified and recorded using this system. This technique uses a dual-spin centrifugation process. The whole blood product is transferred into two sterile 50 mL conical tubes and placed in the centrifuge, IEC Centra-CL2 (Thermo IEC, Needham Heights, MD). The first centrifugation parameter is set to 2,600 rpm for 10 minutes (500 G). After completion of the first spin, the two conical tubes are placed under a biosafety hood for manual extraction of separate blood components. The top fraction of platelet-poor plasma (PPP) is extracted with a pipette and added to a separate 50-mL conical tube. Then, the remaining buffy coat leukocyte layer and red blood cell layer are consolidated into one 50-mL conical tube. The second centrifugation is commenced at 3,400 rpm for six minutes (855 G), followed by the final extraction of the PPP layer. The remaining 5 to 6 mL of the RBC and buffy coat leukocyte layer are resuspended in the conical tube. Lastly, 1.0 mL of the PRP product is pipetted into a 2.0 mL microcentrifuge tube, and the hematology analyzer is used to quantify the final complete blood count [4]. If the final PRP product is above the desired leukocyte count, the PRP is diluted with a minimal fraction of PPP. The final preparation of diluted PRP is then loaded into a sterile dual-syringe system [9]. 

When evaluating the ProofPoint Biologics manual method, our laboratory study of two samples demonstrated a mean platelet increase of 5.2 (±0.28) (Table 1) [2]. The mean total processing time per patient sample was 49 (±1.4) minutes. However, the Proofpoint Biologics manual method is the most time-intensive for the technician of all the POC products illustrated in this review.

**Table 1:** Summary of commercially available point-of-care platelet-rich plasma processing. *Two out of four celling biologics samples produced platelet yields below the baseline. BMC: bone marrow concentrate and PRP: platelet-rich plasma.

Product	Advertised platelet increase (fold)	Laboratory platelet increase (fold; mean ± SD)	Laboratory processing time, minutes (mean ± SD)	Advantages	Disadvantages
ProofPoint	N/A	5.2 (±0.28)	49 (±1.4)	Platelet counts increase consistently by more than four-fold	Manual transfer of blood products using pipette system time-intensive two centrifugation steps
AcCELLerated	4	5.2 (±0.3)	18 (±1.4) (PurePRP® AB60 Pure, Pure Accelerated Biologics, Tequesta, FL) 13.5 (±2.1) minutes (AbsolutePRP®, Emcyte Corporation, Fort Myers, FL)	Shortest processing time offer PRP, BMC, and adipose platelet counts to increase consistently more than four-fold	
Arthrex	2-3	4.2 (±2.0)	15.8 (±6.4)	Decreased processing time representatives available in the operating room	Inconsistent platelet increases
Celling	N/A	2.7 (±0.8)*	29 (±5.2)	Convenient use	Variable results unable to reach four-fold increase device lost many platelets in two trials variability in results with altitude
Terumo	3.62	4.1 (±1.9)	24.43 (±5.56)	Capable of reaching a four-fold concentration of platelets	Significant variability with plasma concentrating longer processing times BMC lost platelets during the demo

AcCELLerated Biologics

AcCELLerated Biologics has multiple PRP compressing kits, such as the PurePRP® AB60 pure and AbsolutePRP®. They advertise these kits as the only current PRP processing kits that can provide multiple processing protocols. Like the Terumo blood system (Terumo, Lakewood, CO), the PurePRP® AB60 pure kit can be used for both PRP and bone marrow aspirate concentrate (BMAC). In both kits, after the patient’s venipuncture, the whole blood is added to their processing container called the ClearVUE Conical Piston. The company highlights this container as having a deep conical shape that is supposed to provide a more concentrated end product and less red cell integration [3]. The PurePRP® AB60 pure is like the Terumo system in that it is a dual-spin processing technique [4]. During the first spin, the blood product is placed in the Platinum Series Centrifuge (Emcyte Corporation, Fort Myers, FL) at the setting of PurePRP® SP SPIN 1 and this is set to 1.5 minutes at 3.8 × 10^3^ revolutions per minute (rpm). After this cycle, the platelet plasma suspension (PPS) is then removed and placed in a concentrating accessory device before being placed back into the Platinum Series Centrifuge device for the second spin cycle, which is then set to 5.0 minutes at 3.8 × 10^3^ rpm. After this, the platelet buffy coat leukocyte layer is separated from the rest of the plasma. The remainder of the solution is then manually shaken in the bottle prior to placement in the final syringe. The AbsolutePRP® system is similar. However, this processing system only requires one spin cycle in the Platinum Series Centrifuge at the AbsolutePRP® setting with one cycle set to 5.0 minutes at 4.4 rpm. 

The AcCELLerated Biologics quotes an increase of four-fold from the baseline for PRP with these kits. Our laboratory study of two samples demonstrated a mean platelet increase of 5.2 (±0.3) fold with PurePRP® AB60 Pure and 4.8 (±0.7) fold with AbsolutePRP® (Table 1) [2]. The mean total processing time per patient sample for PurePRP® AB60 pure was 18 (±1.4) minutes versus AbsolutePRP® at 13.5 (±2.1) minutes. From our results, both AcCELLerated Biologics kits were found to have one of the shortest mean processing times for all POC devices.

Arthrex Angel System

The Angel® cPRP system (Arthrex, Naples, FL) is advertised as a completely closed one-button automation system that can deliver customized PRP concentrations [3]. With this system, 30 different custom processing protocols can be programmed. Like the previous systems described, this system can work for both PRP and BMAC. However, the PRP and BMAC can be mixed with allograft or autograft bone prior to application to a surgical site as a spray, gel, or clot. In contrast to the other systems previously described, the Angel® cPRP system does not require manual separation of the PRP from PPP and RBCs. Rather, after the patient’s venipuncture, the syringe full of the whole blood product (40 ml to 180 ml) is directly injected into the Angel® system centrifuge. A single cycle is commensed and their 3-sensor technology (3ST) automatically separates the whole blood product into PPP, PRP, and RBC by their different wavelength properties into three different syringes. If desired, this process can be repeated two more times for a total of three cycles by simply pressing the “new cycle” button. Approximately one cycle of spin time for 40 mL of whole blood is 15 minutes and 26 minutes for 180 mL of whole blood. 

Arthrex advertises a two- to three-fold increase in platelets in a 10% volume return with their fully automated Angel® cPRP system [2]. Our laboratory study of four samples demonstrated a mean platelet increase of 4.2 (±2.0) with a range of 2.3-6.2 fold (Table 1). The mean total processing time per patient sample was 15.8 (±6.4) minutes. Overall, this automated system seems to decrease the processing time. However, there is variability in the platelet increase.

Celling Biosciences

Celling Biosciences is known for its autologous regenerative therapy (ART) PRP system, which advertises that its concentration systems are able to effectively and efficiently concentrate platelets and growth factors from whole blood through an integrated nano-pore fiber system [4]. The company boasts that efficient concentration yields an ultra-low hematocrit with only one spin. Users can select the desired layer of the centrifuged stack in a controlled manner using their thumb-wheel technology for efficient stack layer extraction. The adjustable flow valve diverts fluid without sterile breaks, allowing for a large collection window. Celling states that their closed system technology promotes sterility [3]. Celling Biosciences offers two products: an ART Two Step, which features a dual-chamber design allowing the user to perform either leukocyte-rich or leukocyte-poor plasma, or the ART PRP Plus, which allows for the ultrafiltration of proteins from platelet-poor plasma.

Our laboratory study of four samples demonstrated variable results regarding final product platelet concentration, with an inability to reach a high concentration of platelets in two of our trials and half the samples demonstrating a platelet level below baseline (2.7 ± 0.8-fold) [2]. The mean total processing time for the four samples was 29 (±5.2) minutes. The manufacturer believes that high altitude may have a significant effect on the device’s ability to concentrate the product. The processing time for this product is decreased at a lower cost than other devices. However, the inconsistency in platelet concentration is a notable limitation.

Terumo blood and cell technologies (BCT)

The Terumo BCT platelet concentrate system is an additional POC product for the preparation of autologous PRP. Following patient venipuncture, the whole blood is added into their processing containers called the SmartPrep® system platelet concentrate process disposable [3]. These are then loaded into their centrifuge system, SmartPrep® multicellular processing system, which is advertised as being capable of processing not only PRP but bone marrow aspirate concentrate (BMAC) and concentrated adipose tissue as well [4]. The Terumo platform works by selectively capturing CD34+ peripheral stem cells while preferentially reducing the granulocyte cell population in the final PRP product. The centrifugation process is a dual-spin process, which takes 14 minutes total. During the first four-minute spin cycle, plasma is separated from red blood cells and captured in a separate chamber. During the next 10-minute spin cycle, platelets are concentrated at the bottom of the plasma chamber. Following centrifugation, two-thirds of the platelet-poor plasma (PPP) is removed and may be discarded. The remaining PPP is then used to resuspend the platelet concentrate to create platelet-rich plasma, which is then ready for clinical use. 

Terumo BCT purports their platelet concentration system to produce a 3.62-fold increase in white blood cells and platelets compared to whole blood (5.83 × 10^3^/mL to 21.09 × 10^3^/mL; manufacturer’s website) [2]. Our laboratory study of seven samples demonstrated a mean platelet increase of 4.1 (±1.9) fold with a range of 0.6-6.2 fold (Table 1). The mean total processing time per patient sample was 24.43 (±5.56) minutes. 

Limitations

One of the main obstacles to discerning the therapeutic benefits of PRP treatment from the myriad of published studies is the insufficient reporting of experimental detail or exact compositions of PRP formulations used. A 2017 systematic review concluded that only 16% of published clinical studies provided any quantitative metrics of the composition of PRP delivered [3]. This conclusion not only makes interpretation of results difficult but precludes comparisons between studies and replication of experiments and clinical trials to confirm results. Thus, due to these inconsistencies in the literature and in companies' variability in reporting data, it is ultimately impossible to recommend a single POC device [8]. Consequently, the clinical efficacy of PRP therapy in orthopedic surgery remains an open and ongoing debate. The need for PRP characterization and standardization for particular indications with demonstrated clinical efficacy is well recognized. In addition, the application of potency assays has also been proposed to help confirm the quality of PRP-derived products and assure their efficacy for the desired indication [2]. Although there is a high demand to understand individual factors present in PRP that play a major determinant in tissue repair, new areas of research are beginning to focus on neutralizing or eliminating certain deleterious factors in PRP to improve the benefit of musculoskeletal repair.

## Conclusions

There is significant variability in the technical features, cost, processing time, and centrifugation parameters of the different commercially available point-of-care devices. Recommending a single device is not possible due to the wide variability in reporting among devices. This review was intended to provide clinicians with information that allows them to decide which combination of features fits their PRP needs best. Additional data is required to make informed decisions on the optimal system for PRP concentration and preparation.
